# The Multifaceted Role of STAT3 in NK-Cell Tumor Surveillance

**DOI:** 10.3389/fimmu.2022.947568

**Published:** 2022-07-05

**Authors:** Agnieszka Witalisz-Siepracka, Klara Klein, Bernhard Zdársky, Dagmar Stoiber

**Affiliations:** ^1^ Department of Pharmacology, Physiology and Microbiology, Division Pharmacology, Karl Landsteiner University of Health Sciences, Krems, Austria; ^2^ Institute of Pharmacology and Toxicology, University of Veterinary Medicine, Vienna, Austria

**Keywords:** STAT3, NK cells, tumor immune surveillance, JAK-STAT, immunotherapy

## Abstract

Signal transducer and activator of transcription 3 (STAT3) is a member of the Janus kinase (JAK)-STAT pathway, which is one of the key pathways contributing to cancer. STAT3 regulates transcription downstream of many cytokines including interleukin (IL)-6 and IL-10. In cancer, STAT3 is mainly described as a tumor promoter driving tumor cell proliferation, resistance to apoptosis, angiogenesis and metastasis and aberrant activation of STAT3 is associated with poor prognosis. STAT3 is also an important driver of immune evasion. Among many other immunosuppressive mechanisms, STAT3 aids tumor cells to escape natural killer (NK) cell-mediated immune surveillance. NK cells are innate lymphocytes, which can directly kill malignant cells but also regulate adaptive immune responses and contribute to the composition of the tumor microenvironment. The inborn ability to lyse transformed cells renders NK cells an attractive tool for cancer immunotherapy. Here, we provide an overview of the role of STAT3 in the dynamic interplay between NK cells and tumor cells. On the one hand, we summarize the current knowledge on how tumor cell-intrinsic STAT3 drives the evasion from NK cells. On the other hand, we describe the multiple functions of STAT3 in regulating NK-cell cytotoxicity, cytokine production and their anti-tumor responses *in vivo*. In light of the ongoing research on STAT3 inhibitors, we also discuss how targeting STAT3 would affect the two arms of STAT3-dependent regulation of NK cell-mediated anti-tumor immunity. Understanding the complexity of this interplay in the tumor microenvironment is crucial for future implementation of NK cell-based immunotherapies.

## Introduction

Natural killer (NK) cells belong to the group 1 innate lymphoid cells and are characterized by the ability to kill virally infected and malignant cells. In contrast to T cells, NK cells do not require major histocompatibility complex (MHC)-dependent priming by antigen presenting cells. The activity of NK cells is regulated by a delicate balance of germ-line encoded activating and inhibitory receptors. Upon recognition of the target cell, an NK cell releases cytotoxic granules for direct cell lysis as well as produces immunomodulatory cytokines (1, 2). In humans, these tasks are fulfilled by different subtypes of NK cells: CD56^bright^CD16^lo/-^ NK cells are main producers of cytokines such as interferon (IFN) γ. In contrast, CD56^dim^CD16^+^ NK cells are highly cytotoxic, but do not produce substantial amounts of IFNγ (3). The inborn ability to lyse transformed cells renders NK cells an attractive tool for cancer immunotherapy with a potentially better safety profile compared to T cells (4, 5). Different approaches to exploit NK cells in immunotherapy are being investigated. These include adoptive transfer of cytokine-induced memory-like NK cells or chimeric antigen receptor NK (CAR-NK) cells. Currently, numerous clinical trials using such approaches are ongoing, but the efficacy of these treatments still needs to be evaluated (5, 6). In this context, understanding the complex processes employed by tumor cells to evade NK-cell immunity is crucial. These escape mechanisms include transcriptional downregulation and shedding of ligands for NK-cell activating receptors, upregulation of inhibitory ligands, as well as immune suppressive signals derived from the microenvironment (7–11). Signal transducer and activator of transcription 3 (STAT3) is constitutively activated in various cancers and plays a pivotal role in regulating all of these processes and thereby mediates the crosstalk between the tumor microenvironment and immune cells (12).

**Figure 1:**
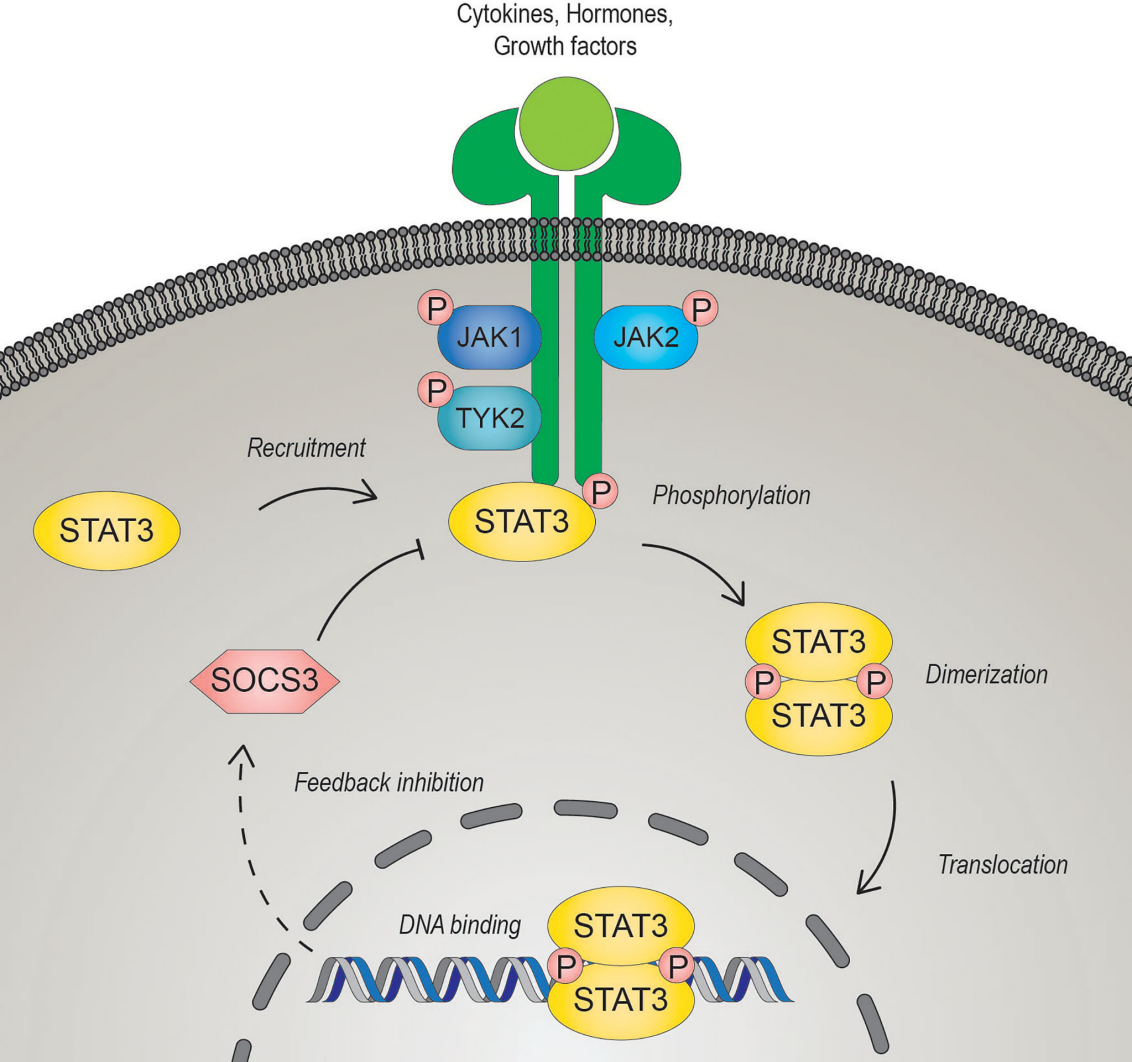
The JAK-STAT3 signaling pathway. Signal transducer and activator of transcription 3 (STAT3) is activated upon binding of diverse cytokines, hormones, or growth factors to their cognate receptors. Ligand-bound receptors undergo conformational changes leading to the activation of the Janus kinases (JAK). Activated JAKs trans- and/or auto- phosphorylate each other and the cytoplasmic domain of the receptor, enabling STAT3 to bind *via* its SRC homology 2 (SH2) domain. JAK-mediated phosphorylation of a conserved C-terminal tyrosine residue of STAT3 induces dimerization of phosphorylated STAT3 and the subsequent translocation to the nucleus to regulate gene transcription. STAT3 induces transcription of suppressor of cytokine signaling 3 (*SOCS3*), which can act as a negative regulator by interacting competitively with the receptor.

STAT3 is a member of the Janus kinase (JAK)-STAT signaling pathway, which coordinates central cellular mechanisms including differentiation, development, proliferation, immune function, or apoptosis (13, 14). The alternatively spliced STAT3 isoforms, full-length STAT3α and C-terminally-truncated STAT3β, have opposing function during tumor development. While STAT3α promotes tumor growth, STAT3β was identified as a tumor suppressor and favorable prognostic marker in cancers of different origin (15, 16). Mechanistically, JAK-STAT3 signaling is activated by diverse growth factors, peptide hormones and all interleukin (IL)-6-type cytokines including IL-6, IL-11, IL-27, IL-31, leukemia inhibitory factor (LIF), oncostatin M (OSM), ciliary neurotrophic factor (CNTF) neuropoietin (NP), cardiotrophin-1 (CT-1) and cardiotrophin-like cytokine (CLC) (17–19). IL-6 family cytokines, except for IL-31 which exerts its effects through IL-31 receptor α, induce signaling *via* binding to either a glycoprotein 130 (gp130) receptor β- subunit hetero- or homodimer (19, 20). Ligands bind to their cognate receptors, which undergo a conformational change, and induce subsequent activation of receptor associated JAKs (JAK1, JAK2, JAK3 and tyrosine kinase 2 (TYK2)) by autophosphorylation and/or transphosphorylation. The JAK-induced tyrosine phosphorylation of the receptor provides a docking-site for the SH2 domain of STAT3, which in turn gets phosphorylated on tyrosine 705 by JAKs (21). Activated STAT3 forms anti-parallel to parallel homo- or heterodimers with other STATs, is released from the receptor and translocates into the nucleus through interaction with importin-β1 (22, 23). To control gene expression, activated STATs target palindromic consensus sequences located in promoter and enhancer regions and in the first introns of target genes (24). Negative regulation of STAT3 occurs in the nucleus through antagonization by PIAS (protein inhibitor of activated STAT), an E3 SUMO-protein ligase, or at the receptor by SOCS (suppressor of cytokine signaling) E3 ubiquitin ligases (25, 26) (**Figure 1**). The transcriptional activity of STAT3 can be further regulated by phosphorylation at serine 727 (Ser727) mediated by mTOR, p38, ERK and other serine/threonine kinases. However, the exact effects of Ser727 phosphorylation have to be put in a cellular and/or promoter dependent context. Phosphorylated Ser727 promotes association with different transcription co-factors and thus activates or diminishes transcriptional responses of STAT3 (27). Moreover, it can also drive the mitochondrial metabolic activity of STAT3 and augment the electron transport chain (28). Activated STAT3 in cancer cells contributes to oxidative and glycolytic phosphorylation, survival, epithelial-to-mesenchymal transition, proliferation, metastasis, and radiation- and chemotherapy resistance (29, 30). Due to its central contribution to several hallmarks of cancer and its association with poor clinical prognosis, STAT3 represents a promising therapeutic target for cancer therapy (14, 31, 32).

Inhibition of STAT3 signaling is currently explored in many clinical trials for solid and hematopoietic tumors. The direct approaches to specifically inhibit STAT3 include small molecules and decoy oligonucleotides (33). The most successful small molecule STAT3 inhibitor Napabucasin (BBI-608), which selectively binds to the DNA-binding domain of STAT3, has reached phase III trials for advanced colorectal cancer and provided excellent results as monotherapy (34). Another small molecule, TTI-101, targets the receptor binding site within the SH2 domain of STAT3 to block its recruitment and activation (35). Phase I clinical trials in advanced solid cancers including breast cancer are ongoing (NCT03195699, NCT05384119). The SH2 domain is also targeted by other small molecules, OPB- 51602, OPB-31121, OPB-111077, which are undergoing phase I/II clinical trials for solid and in case of OPB-51602 also hematopoietic tumors (reviewed in (36)). The antisense oligonucleotide, AZD9150, which is designed to target *STAT3* mRNA (37), has until now reached phase I/II trials for different advanced solid cancers (e.g. NCT01839604; NCT01839604) (33). Although the specificity and potency of such antisense oligonucleotides is very promising, they face problems of efficient penetration of solid tumors and fast degradation (33). To the best of our knowledge, the STAT3 inhibitors currently tested in clinical trials have not been thoroughly studied in the context of NK cell anti-tumor responses. Here, we summarize the current knowledge on how STAT3 contributes to NK-cell fitness and tumor cell evasion from NK cells, and speculate on how targeting STAT3 may affect NK-cell tumor surveillance.

## STAT3 in Tumor Cells – The Driver of Immune Evasion From NK Cells

### Regulation of NK-Cell Receptor Ligands

NK cells exhibit cytolytic activity towards cells that overexpress ligands for activating receptors and/or lack the expression of MHC class I and other ligands, recognized by inhibitory receptors. In healthy cells, the ligands for activating receptors are absent or expressed at very low levels. Transformed cells upregulate these ligands and become sensed by NK cells as ‘danger’ (1, 38). The activating NK-cell receptor natural killer group 2D (NKG2D) served as a paradigm in studying this mechanism. In humans, NKG2D binds to MHC class I polypeptide-related sequence A (MICA) and B (MICB) and UL16-binding proteins (ULBP1-6) (39–42). In mice, NKG2D ligands comprise of the RAE1 family (α-ϵ), H60 (a -c), and MULT1 (43–45). Upon binding of a ligand to NKG2D, the co-stimulatory molecule DAP10 is activated and the release of cytotoxic granules is induced (40). Natural cytotoxicity receptor (NCR) NKp30 recognizes the B7-H6 molecule on transformed cells and induces NK-cell activation (46). Other receptors of this family are NKp44 and NKp46, which recognize heterogenous ligands including viral and bacterial proteins (47). Further activating NK-cell receptors function more as amplifiers of NK-cell activation triggered by NKG2D or NCRs (48). An important example is DNAM-1 and its corresponding ligands CD112 and CD155 often overexpressed on tumor cells (49, 50).

NKG2D is crucially involved in NK cell-mediated tumor surveillance and is one of the best studied receptors in this context. Mice deficient in NKG2D show strong defects in immune surveillance of epithelial and lymphoid tumors (51). In line, high expression of NKG2D ligands in leukemic patients correlates with better survival (52). Absence of NKG2D ligands is also a feature of leukemic stem cells, which allows them to escape NK-cell surveillance in acute myeloid leukemia (AML) *in vivo* models (53). Tumor cells evade NKG2D-mediated recognition by downregulation or shedding of the ligands. Not only does it allow to hide from NK-cell cytotoxicity, but also leads to desensitization of NKG2D-mediated NK-cell activation. High levels of shed NKG2D ligands result in downregulation of NKG2D-mediated signaling (54, 55).

STAT3 has been implicated in direct transcriptional repression of NKG2D ligands. Bedel et al., revealed that inhibition or knockdown of STAT3 in the colorectal cancer cell line HT29 leads to stronger activation of NK cells and therefore killing of tumor cells in an NKG2D-dependent manner. Further, they could show that STAT3 directly binds to the *MICA* promoter, repressing its transcription (56). A similar mechanism has been described in multiple myeloma (MM) cell lines. Upon treatment with glycogen synthase kinase 3 (GSK-3) inhibitor, MM cell lines showed decreased STAT3 activation and reduced STAT3 binding to the *MICA* promoter. The effects corresponded to enhanced sensitivity of treated cell lines to NK cell-mediated lysis. Importantly, GSK-3 inhibition had no effect on *MICA* expression in cell lines with constitutively active STAT3. In line, the GSK-3 inhibitor was not able to reduce the activation level of constitutively active STAT3. This strongly suggests that GSK-3-induced susceptibility of MM cells to NK cells is greatly dependent on inhibiting STAT3 activation (57). In another study using colorectal cancer cells, GSK-3 inhibition significantly upregulated NKG2D ligands and increased their sensitivity to NK cells. However, in this context the dependence on STAT3 remains to be elucidated (58).

The correlative observations of low STAT3 activity and/or expression and high NKG2D ligands surface levels have also been made in other cancer entities. The adriamycin-resistant chronic myeloid leukemia (CML) cell line K562 is killed more efficiently by NK cells upon treatment with a STAT3 inhibitor and shows an upregulation of MICA and ULBP2 (59). STAT3 inhibition or silencing also enhances ULBP2 expression in parental K562 cells (60). In AML cell lines, an inverse correlation between phosphorylated STAT3 (pSTAT3) levels and MICA expression was observed after rapamycin (61) or decitabine treatment (62) but the mechanism behind it remains unclear. In hepatocellular carcinoma cell lines, a STAT3 decoy resulted in upregulation of NKG2D ligands and increase of NK cell-mediated killing (63). A layer of complexity is added by the fact that in human gastric adenocarcinoma cell lines inhibition of STAT3 results in upregulation of MICB on the cell surface as well as of the soluble ligands. This implies a potential desensitization of NK cells driven by inhibition of STAT3 (64). Although all the mentioned studies point towards a similar effect of STAT3 inhibition or silencing, the interpretation is limited, as all of the experiments where only performed *in vitro*. A robust *in vivo* xenograft model of STAT3-deficient tumor cell lines with adoptive transfer of human primary NK cells would be necessary to further elucidate the impact of STAT3 on NKG2D-mediated NK-cell surveillance.

The missing-self hypothesis, formulated in the 1980s, states that NK cells kill those cells that do not express sufficient levels of MHC I. In line, several classes of inhibitory receptors were discovered, which unleash NK-cell cytotoxicity upon downregulation of MHC I on the target cells (65, 66). These include the Ly49 family in mice and the KIR family in humans (67–69). Ly49 and KIRs sense the levels of conventional MHC I, therefore, tumor cells that downregulate MHC I molecules to escape T cell responses become targets for NK cells (70, 71). In a mouse model of carcinogen-induced Non-Small Cell Lung Cancer (NSCLC), epithelial cell-specific knockout of *Stat3* led to downregulation of MHC I on transformed epithelial cells. This rendered the emerging cancer cells more susceptible to NK cell-mediated lysis (72).

### Regulation of Tumor Microenvironment

STAT3 is considered as a driver of an immune suppressive tumor microenvironment. STAT3 activation is associated with high expression of tumor promoting cytokines and growth factors such as IL-10, transforming growth factor (TGF)-β and vascular endothelial growth factor (VEGF)-A. The majority of STAT3-dependent effects in the tumor microenvironment are described in the context of T cells, macrophages or dendritic cells that have been extensively reviewed by others (12, 73). However, the soluble immune suppressive modulators present in tumor microenvironment not only suppress the function of NK cells, but also impair their infiltration into the tumor (13) or even confer a switch towards pro-tumorigenic, VEGF-A-producing NK cells (74–77). The IL-10/STAT3 axis directly drives VEGF-A expression in these cells (77). TGF-β in the microenvironment also drives the conversion of cytotoxic NK cells (CD49a^-^CD49b^+^EOMES^+^) into ILC1 (CD49a^+^CD49b^-^EOMES^int^), which lose the ability to control the tumor growth (78).

In the previously mentioned, carcinogen-induced NSCLC model with epithelial cell-specific *Stat3* knockout, the tumor microenvironment is enriched for proinflammatory cytokines which might contribute to enhanced NK-cell responses against the tumor (72). In support of this finding, hepatocellular carcinoma cells treated with STAT3 decoy secrete higher levels of IFNs and lower levels of immune suppressive TGF-β. Upon culture in conditioned medium derived from hepatocellular carcinoma cells pre-treated with STAT3 decoy, NK cells showed a more activated phenotype with higher expression of IFNγ, granzyme and perforin (63). In line, STAT3 in a murine melanoma cell line was shown to inhibit the expression of proinflammatory cytokines such as TNFα and IL-12 and the chemokine CCL5. Inhibition of STAT3 signaling was associated with increased levels of CCL5 and thereby enhanced lymphocyte infiltration into the tumor (79). Importantly, the conclusions were made in melanoma model overexpressing the alternatively spliced, truncated isoform of STAT3 – STAT3β, which was believed to have dominant negative functions over the full length isoform (80). However, several studies have shown that STAT3β is transcriptionally active and drives expression of its unique target genes (81–83). The potential of STAT3β to drive cytokines and chemokines that support the immune system is in accordance with the tumor suppressive potential of STAT3β-overexpressing macrophages in breast cancer (84).

Interestingly, in BCR-ABL-driven lymphoma, the deletion of STAT3 has opposite effects to those described above. STAT3-deficient B cell lymphoma shows decreased expression of proinflammatory cytokines, e.g. TNFα and chemokine CCL5. This is paralleled by a lower abundance of NK cells in the tumors. Transplantation of the lymphoma cells lacking STAT3 into mice harboring NK cells results in accelerated tumor growth, but the difference is lost in immune-deficient mice. The study postulates that targeting STAT3 in BCR-ABL-driven malignancies might impair NK-cell surveillance (85).

**Figure 2:**
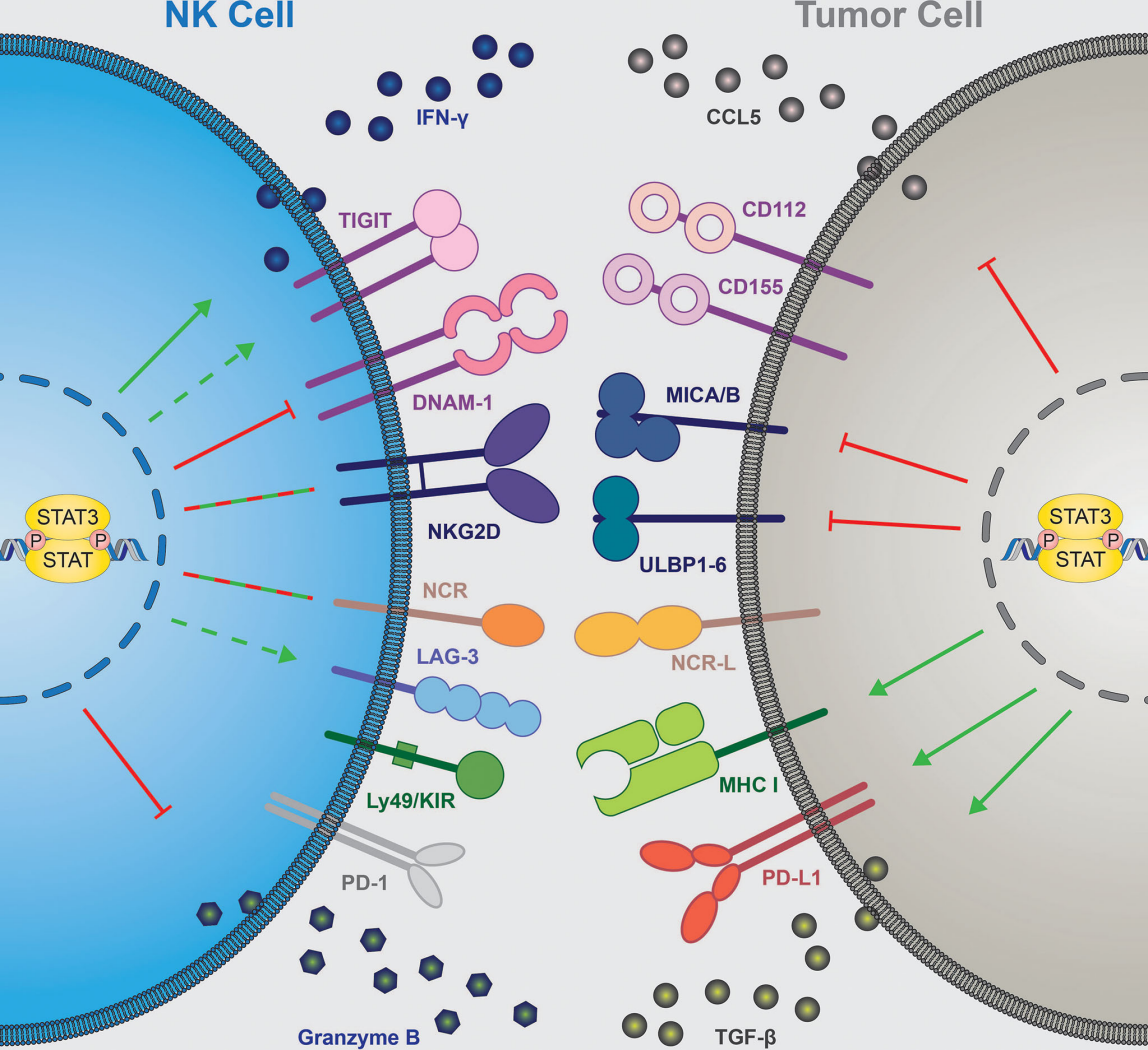
STAT3 contribution to NK cell-mediated tumor immune surveillance. NK cell-intrinsic STAT3 (**left**) inhibits expression of granzyme B and DNAM-1 (
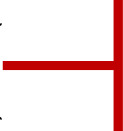
 **)**, increases IFNγ secretion (**
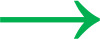
**) and seems to upregulate TIGIT and LAG-3 (
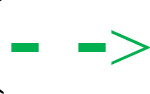
), while the effect on NCRs and NKG2D expression remain context dependent **(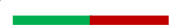)**. Tumor cell-intrinsic STAT3 (**right**) inhibits expression of NKG2D ligands (MICA/B, ULBPs) and NK-cell attracting chemokine CCL5 (
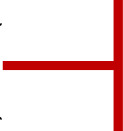
). STAT3 in tumor cells upregulates surface expression of MHC I and PD-L1 molecules and secretion of immune suppressive TGF-β (**
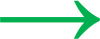
**). NK, natural killer; IFN, interferon; DNAM-1, DNAX accessory molecule; NKG2D, NK-cell receptor natural killer group 2D; NCR, natural cytotoxicity receptor; KIR, killer-cell immunoglobulin-like receptor; CCL5, C-C motif chemokine ligand 5; CD, cluster of differentiation; MICA/B, major histocompatibility complex class I-related sequence A/B; ULBP, UL16-binding protein; MHC I, major histocompatibility complex I; TGF-β, transforming growth factor β, STAT, signal transducer and activator of transcription. TIGIT, T cell immunoreceptor with Ig and ITIM domains; LAG-3, lymphocyte-activation gene 3; PD-(L)1, programmed cell death ligand/protein 1.

In summary, STAT3 activity is critically implicated in determining the outcome of cancer immunity by orchestrating the release of immunomodulating cytokines. In the majority of cases, inhibition of STAT3 signaling switches the tumor microenvironment towards immune activation (**Figure 2**, right).

## STAT3 in NK Cells – The Versatile Modulator of NK Cell Responses

STAT3 is activated in NK cells by a variety of cytokines, including type I IFNs, IL-2, IL-6, IL-10, IL-12, IL-15, IL-21 and IL-27, with diverse effects on NK-cell activation (86–91). NK cell-intrinsic roles of STAT3 have been analyzed in *Stat3^fl/fl^ Ncr1-iCre* mice lacking STAT3 in NKp46+ cells. *Stat3* deletion does not affect NK-cell development, numbers and maturation. Furthermore, NK cell-intrinsic loss of STAT3 does not impact on proliferation of NK cells in *Stat3^fl/fl^ Ncr1*-iCre mice (87). However, knockdown of STAT3 in the human NK-cell line NK-92 is associated with a decreased proliferation rate, correlating with reduced *cyclin D1* expression, and overexpression of STAT3 enhances human NK-cell expansion (92). In line, IL-21, which primarily activates STAT3 in NK cells, promotes human NK-cell expansion associated with an increased telomere length (93–95).

Effects of cancer cell-extrinsic STAT3 deficiency on anti-tumor immunity have initially been reported in an inducible STAT3 knockout mouse model (*Stat3^fl/fl^ Mx1*-Cre mice) (96). Both *Stat3^fl/fl^ Mx1*-Cre and *Stat3^fl/fl^ Ncr1*-iCre mice demonstrated that lack of STAT3 enhances NK cell-mediated surveillance in different transplantable tumor models (87, 96). Treatment with the small-molecule STAT3 inhibitor CPA7 boosts anti-tumor responses against the subcutaneously injected urothelial carcinoma cell line MB49, which is largely dependent on T cells with a partial involvement of NK cells (96). NK cell-intrinsic STAT3 deficiency is sufficient to increase surveillance of melanoma and leukemia cell lines (87). Overall, these data provide evidence that STAT3 suppresses the anti-tumor activity of NK cells (13, 87, 96). The enhanced cytotoxicity of NK cells upon loss of STAT3 goes along with increased levels of the cytotoxic effector molecules perforin and granzyme B (87, 88).

STAT3 is also involved in regulating expression of activating NK-cell receptors. STAT3 decreases DNAM-1 expression on NK cells. Elevated DNAM-1 levels upon *Stat3* deletion contribute to enhanced killing of DNAM-1 ligand-expressing tumor cells, e.g. B16F10 melanoma cells (87). NK cells show a STAT3-dependent upregulation of NKG2D surface levels in response to IL-10 and IL-21 stimulation, associated with enhanced NK-cell degranulation (88, 95, 97). While NK cell-intrinsic loss of STAT3 does not suffice to impact on NKG2D expression, STAT3-deficiency in the entire hematopoietic system (*Stat3^fl/fl^ Tie2*-Cre mice) causes a reduction of NKG2D levels (87, 95). In contrast to a potential negative regulatory role of STAT3 on DNAM-1 expression in murine NK cells (87), these results indicate that STAT3 enhances NKG2D expression (95). However, another report demonstrated that IL-2-activated human NK cells display decreased NKG2D levels upon IL-21 stimulation (98). In addition, STAT3 activation by IL-6 and IL-8 produced by tumor cells has been reported to decrease levels of NKG2D and NKp30 on NK cells (91). Therefore, the impact of STAT3 on the regulation of NKG2D might vary depending on the specific upstream stimuli, signaling pathways and additional STAT proteins involved (13, 88). Similar to NKG2D, STAT3 has also been reported to bind to the promoter and drive the transcription of N*CR1* gene encoding NKp46 (92, 95).

In line with the suppressive effect of STAT3 on NK-cell functionality reported in the murine system (87), tumor-derived cytokines, such as IL-6 and IL-8, impair human NK-cell function in a STAT3-dependent manner (91). On the contrary, another study found a positive correlation between STAT3 levels and expression of cytotoxic effector molecules and cytokines in human NK cells (92). STAT3 levels are reduced in NK cells from chronic hepatitis B virus (HBV) patients, which is associated with lower degranulation and IFNγ production (92). Early cytokine production, including IFNγ, in IL-15 primed human NK cells also requires STAT3 (99). An involvement of STAT3 in the regulation of IFNγ production has also been described in murine NK cells, where STAT3 directly binds to the *Ifng* promoter and contributes to cytokine-induced IFNγ production (87). Besides cytotoxic activity and production of proinflammatory cytokines, NK cells also produce immunosuppressive cytokines and have immunoregulatory functions (100, 101). STAT3 also impacts on these functions. For example, the immunosuppressive cytokines IL-10 and TGF-β are upregulated in a STAT3-dependent manner in tumor-infiltrating NK cells that are positive for the immune checkpoint CD73 (12, 102).

Hypoxia is an important feature of solid tumors that is associated with immune suppression and escape (103). Indeed, hypoxia represses NK-cell cytotoxicity by induction of SHP-1 expression, which in turn reduces STAT3 activation (104). STAT3 also interacts with one of the main players driving hypoxic response - hypoxia inducible factor 1α (HIF-1α) (105). In IL-15 primed human NK cells, HIF-1α responses rely on STAT3 (99). In line, STAT3 induces HIF-1α-mediated upregulation of miR-224, which is paralleled by reduced NKp46 expression and a dampened NK cell-mediated killing of prostate cancer cells (12, 106, 107). The exact underlying mechanisms how STAT3 activity contributes to hypoxia-driven effects on NK-cell cytotoxicity remain to be determined.

Altogether, STAT3 has complex effects on NK-cell activity, including the expression of cytotoxic granule proteins, cytokines and NK-cell receptors (**Figure 2**, left). Whether STAT3 has an overall beneficial or detrimental effect on NK-cell function appears to be context-dependent (13, 87, 92, 95). STAT3 induces its own negative feedback regulation, including the upregulation of SOCS3 (**Figure 1**). SOCS3 suppresses NK cells, as loss of SOCS3 enhances NK-cell proliferation and cytotoxicity (108). The effects of SOCS3 on STAT3 activation might depend on the upstream stimuli, which are likely to be differently susceptible to SOCS3-mediated inhibition (109, 110). This could contribute to the context-dependent effects of STAT3 in NK cells. As mentioned above, cancer cell-intrinsic alternatively spliced STAT3 isoforms have opposite roles in driving tumor progression. Since STAT1 isoforms differentially affect NK-cell functionality (111), it is attractive to speculate that also STAT3α and β have non-redundant functions in regulating NK cells. Analyzing the consequences of STAT3α or β deficiency in NK cells might improve our understanding of STAT3-dependent effects.

## STAT3 Mutations in NK Cells – Insights From Patients

Another level of evidence for a crucial role of STAT3 in NK-cell biology stems from studies analyzing NK cells in patients with STAT3 mutations. Heterozygous germline STAT3 loss-of-function (LOF) mutations are found in autosomal dominant hyper IgE syndrome (HIES) patients, which display immunological deficiencies with increased susceptibility to infections linked to impaired STAT3-regulated T helper 17 (Th17)-mediated immune responses and B cell function (112–116). NK cells from HIES patients harboring STAT3 LOF mutations have decreased NKG2D levels (95). This might be associated with impaired NK cell function, however a thorough functional characterization of NK cells from HIES patients has not been published. Apart from LOF mutations, germline and somatic activating mutations of STAT3 have been described in humans with different disease characteristics (112–115, 117). Germline STAT3 gain-of-function (GOF) mutations are associated with diverse clinical manifestations, including immunodeficiencies and autoimmune diseases (112, 118–122). Haapaniemi et al. reported that NK-cell numbers are reduced in patients with germline STAT3 GOF mutations, while maturation and functionality of NK cells are unaffected (120). However, another study did not find reduced NK-cell numbers (123), indicating that the impact of STAT3 GOF mutations on immune cells, including NK cells, varies between patients (122).

An oncogenic potential of STAT3 in NK cells has been indicated by the discovery of somatic STAT3 GOF mutations, predominantly within the SH2 domain, in a subset of patients with different NK-cell malignancies, including chronic lymphoproliferative disorder of NK cells (CLPD-NK), aggressive NK-cell leukemia (ANKL) and extranodal NK/T-cell lymphomas of nasal type (NKTCL) (124–135). Apart from STAT3 GOF mutations, enhanced STAT3 phosphorylation can also be observed in NK-cell malignancies by other means, including activation of upstream JAKs or reduced expression of negative regulators of STAT3 (124, 127, 131–133, 136–139). Pro-tumorigenic effects of STAT3 on NK cells are linked to its role in proliferation and survival (133, 136, 140–142). IL-10 mediated STAT3 activation as well as somatic STAT3 GOF mutations increase expression of MYC and thereby drive metabolic activation of ANKL cells, fueling leukemia cell survival and proliferation (131). To the best of our knowledge, the effects of somatic STAT3 GOF mutations on the functionality of malignant NK cells have not been directly tested. Interestingly, STAT3 GOF mutations in CLPD-NK patients correlate with a cytotoxic CD16^hi^CD57-phenotype and a more symptomatic disease, characterized by anemia and severe neutropenia (127, 134, 135, 143).

## STAT3 – A Potential Target to Enhance Immune Checkpoint Inhibitor Therapy?

Immune checkpoint inhibitors are one of the most successful approaches of immunotherapy. By targeting the inhibitory ligands (programmed cell death ligand 1 - PD-L1) or inhibitory receptors (programmed cell death protein 1 - PD-1, cytotoxic T-lymphocyte-associated protein 4 - CTLA-4) with monoclonal antibodies, the immune response against tumors can be unleashed (144). It is clear that T cells are the main drivers of immune checkpoint inhibitor responses, but a role of NK cells herein has been proposed (145). Several reports find PD-1-dependent effects of NK cells in specific tumors including MM (146), Kaposi sarcoma (147), Hodgkin lymphoma (148) and head and neck cancer (149). Other studies indicate that the expression of PD-1 in NK cells is minor or neglectable (150) and the exact function of NK cells in anti-PD-1/PD-L1 therapy remains a matter of debate (151). Importantly, vast evidence indicates the direct involvement of STAT3 in driving PD-L1 and PD-L2 expression in tumor cells (132, 152–154). For example, in T cell lymphoma STAT3 is required for induction of *PD-L1* transcription by directly binding its promoter (153). Therefore, combinatorial inhibition of STAT3 and PD-L1/PD-1 axis has been explored as an attractive approach. Encouraging results from preclinical studies (33, 155, 156) led to first clinical trials combining STAT3 inhibitor (BBI-608) with anti-PD-L1 therapies in metastatic colorectal carcinoma (NCT03647839, NCT02851004) or STAT3 targeting antisense oligonucleotide (AZD9150) with anti-PD-L1 therapy in NSCLC (NCT03334617) and other solid tumors. It remains unclear whether NK cells contribute to this combination therapy outcome. Xu et al. suggested that *in vitro* combination of PD-L1 and STAT3 inhibition enhances NK-cell cytotoxicity against prostate cancer cell lines, but the *in vivo* relevance still needs to be elucidated (157).

Novel immune checkpoint molecules are currently emerging and some have entered clinical trials or have recently been approved. In contrast to the above described PD-1 and CTLA-4, the expression of novel checkpoints: T cell immunoreceptor with Ig and ITIM domains (TIGIT), lymphocyte-activation gene 3 (LAG-3) and T cell immunoglobulin and mucin-domain-containing-3 (TIM-3) is clearly shared between T and NK cells (158–161). The exact role of STAT3 in the regulation of these checkpoints has not been addressed in NK cells. In T regulatory cells, TIM-3 is strongly downregulated upon STAT3 inhibition suggesting a potential dependency of TIM-3 expression on STAT3 in other lymphocytes (162). It is attractive to speculate that LAG-3 expression in NK cells might be enhanced by STAT3 signaling. IL-12, which predominantly signals *via* STAT4 and STAT3, was shown to upregulate LAG-3 expression in NK cells (163). In line, STAT3 inhibition together with blockage of the STAT3-activating cytokine IL-6, downregulated TIGIT expression in the human NK92 cell line. Combination of TIGIT checkpoint inhibitor with blockage of IL-6R and STAT3 enhanced cytotoxicity of NK92 cells towards prostate cancer cells (164). Although further investigations are essential, both studies suggest a potential synergism between STAT3 targeting molecules and novel checkpoint inhibitors in driving NK responses.

## Conclusion and Outlook

STAT3 regulates immune evasion from NK cells on several different levels. It helps tumor cells to hide from NK cells by downregulating activating ligands, drives an immune suppressive environment, which in turn limits the chemoattraction and activity of NK cells, and intrinsically regulates NK-cell responses (**Figure 2**). Somatic GOF mutations in STAT3 have an oncogenic potential in NK cells underlining its key role in NK-cell biology. It remains unclear why the effects of STAT3 on NK-cell biology are so complex and context dependent. The discrepancy between some findings in human and mouse NK cells might come from *in vitro* cultures of human NK cells that do not reflect the situation *in vivo* with different cytokines present in the tumor microenvironment in the mouse models. Moreover, one cannot exclude that the results obtained using mice with STAT3-deficient NK cells are influenced by compensatory mechanisms, including upregulation of STAT5, which is a key driver of NK-cell survival and functionality (77, 165, 166). Not only can the STATs compensate for each other (23, 167) but also crosstalk to other signaling pathways (168). This adds another layer of complexity in understanding the role of STAT3 in NK-cell anti-tumor responses.

Inhibition of STAT3 signaling is currently explored in many clinical trials for solid and hematopoietic tumors. The direct approaches have reached clinical trials but achieving specificity over other STAT family members remains challenging (33, 169, 170). Based on the current evidence, STAT3 inhibition might not only impair tumor cell survival but also enhance their recognition by NK cells. Moreover, targeting NK cell-intrinsic STAT3 could unleash their anti-tumor responses in some tumor models (87), while the consequences on other aspects of NK-cell functionality are difficult to predict. At the moment, there is an unmet need to understand the effects of STAT3 inhibitors on NK-cell anti-tumor responses *in vivo* to be able to foresee the clinically relevant consequences.

It is well appreciated that combination therapies enhance the efficacy and reduce resistance compared to monotherapies. This has triggered extensive attempts in combining STAT3 inhibitors with other drugs (33). A new avenue in immunotherapy is opened by combinations of STAT3 inhibitors explored with the emerging immune checkpoint inhibitors. Importantly, targeting STAT3 in the immune system might have complex systemic effects ranging from autoimmunity to immunodeficiency as indicated by phenotypes of patients with mutations in STAT3 (112–116). In summary, targeting STAT3 might be an attractive approach in restoring NK-cell anti-tumor immunity but needs to be carefully evaluated in different tumor types and biological contexts.

## Author Contributions

AW-S, KK and BZ designed the concept and wrote the manuscript. DS designed the concept and critically reviewed the manuscript. All authors contributed to the article and approved the submitted version.

## Funding

This work was supported by the Austrian Science Fund (FWF) under grant P32693 to DS and the Austrian Academy of Sciences (ÖAW) (DOC scholarship to KK).

## Conflict of Interest

The authors declare that the research was conducted in the absence of any commercial or financial relationships that could be construed as a potential conflict of interest.

## Publisher’s Note

All claims expressed in this article are solely those of the authors and do not necessarily represent those of their affiliated organizations, or those of the publisher, the editors and the reviewers. Any product that may be evaluated in this article, or claim that may be made by its manufacturer, is not guaranteed or endorsed by the publisher.
